# Opportunities and challenges for formalizing community-based palliative care volunteer work: An ethnographic study

**DOI:** 10.1177/26323524261453401

**Published:** 2026-08-05

**Authors:** Hanum Atikasari, Natashe Lemos Dekker, Annemarie Samuels

**Affiliations:** 1 Institute of Cultural Anthropology and Development Sociology, Leiden University, The Netherlands; 2 Department of Society Studies, Faculty of Arts and Social Sciences, Maastricht University, The Netherlands; 3 Department of Anthropology, University of Amsterdam, The Netherlands

**Keywords:** Indonesia, community-based healthcare, ethnography, formalization, palliative care, public health, volunteer

## Abstract

**Background:**

The provision of community-based palliative care is widely recognized as an effective strategy for improving the end-of-life care. Given that both national and international policies increasingly advocate the integration of community-based palliative care programs into broader public health systems, those programs that are often rooted in grassroots movements and mostly run through informal volunteer networks are expected to move toward greater formalization.

**Objectives:**

This study aims to understand how individuals involved in community-based palliative care perceive the movement toward formalizing volunteer work and to identify the opportunities and potential challenges of formalizing community-based palliative care.

**Design:**

This study employed an ethnographic design grounded in qualitative research methods.

**Methods:**

Data were collected during 8 months of ethnographic fieldwork in Jakarta, the capital of Indonesia, in 2022, with a follow-up visit in 2023. The fieldwork entailed prolonged participant observation, focus group discussions, and semi-structured interviews with 14 palliative care volunteers and 6 NGO workers.

**Results:**

This study reveals that palliative care volunteers hold diverse perspectives on the definition of formalization, ranging from the introduction of visible markers (e.g., uniform and identity cards), to the establishment of institutional features (e.g., financial incentives, recognition within the health system, and administrative structures). The opportunities associated with formalization include strengthening volunteers’ legitimacy and recognition in the health system and increased financial rewards. The challenges include concerns about its impact on the spiritual and altruistic nature of volunteer work, increased bureaucratic responsibilities, changes to existing care practices, and concerns about navigating the language of palliative care.

**Conclusion:**

As community-based palliative care becomes increasingly formalized, it is important to understand how this process unfolds across different regions and cultural contexts, and to identify strategies to preserve the unique strengths of volunteer-based care as these formal structures are introduced.

## Introduction

Community-based approaches to palliative care have gained growing attention worldwide as both an alternative and a complement to hospital- and hospice-based models, strengthening support for individuals at the end-of-life or with serious illness.^1–4^ One example of a particularly influential model is Neighbourhood Networks in Palliative Care (NNPC), developed in Kerala, India.^4–7^ Established in 1993, NNPC has become a globally recognized model for delivering community-led palliative care, particularly in resource-constrained settings.^4,8,9^ It is frequently cited for its innovative, cost-effective, and resource-efficient approach to care.^3,4,9,10^ In the Global North, the format of “compassionate communities” has been developed as a public health approach to end-of-life and palliative care, and this model is now active in 19 countries across the Americas, Europe, and the Asia-Pacific region.^1,6,11^

Unlike other health issues (e.g., dengue prevention or maternal and child health programs) that have long been integrated into global health agendas, national policies, and community health systems, community-based palliative care remains relatively new within the public health framework.^12,13^ Its incorporation into both global and national health strategies has been relatively slow.^13,14^ Nevertheless, a growing body of research emphasizes the essential role of palliative care within public health interventions and increasingly recognizes it as a significant public health concern^15–19^ given the serious burden of health-related suffering affecting many countries, especially in the Global South.^20,21^ However, it was not until the 2018 Declaration of Astana that the World Health Organization explicitly affirmed its commitment to strengthening global palliative care efforts—particularly by supporting the integration of community-based palliative care services into primary healthcare systems.^22^

Community-based approaches to palliative care take diverse forms around the world. Some models are highly formalized, while others benefit from informal structures.^7,23–25^ Many of these less formal initiatives are sustained by volunteer efforts. In the Global South, these often emerge from grassroots movements as they are deeply rooted in the cultural value of mutual care within the community.^23,26^ With recent international and national policies emphasizing the integration of palliative care into health systems,^22,27^ it is likely that community-based models—particularly those that rely on informal volunteer networks—will move toward greater formalization, as has been observed for other public health issues.^25,28–33^ Formalization refers to the extent to which an organization defines and enforces rules, regulations, and policies that guide member behavior by institutionalizing and standardizing its procedures and practices.^34^ Analysis of the formalization of other community-based health programs shows that formalization is largely driven by increasing expectations of efficiency, transparency, and accountability, alongside the introduction of institutional forces such as enhanced monitoring systems, mandatory reporting requirements, legal and regulatory obligations, and dominant management practices.^28,32^

However, previous scale-ups of global health interventions suggest that the formalization of community-based programs may have unintended consequences and cause tensions, potentially in terms of compromising the principles of volunteerism and care.^32,35,36^ Studies in Western Kenya^32,37^ and Mozambique,^36,38^ for example, highlight that the formalization of the role of health volunteers who provide home-based care for people living with HIV/AIDS has transformed the practice of care by redefining it narrowly as clinically oriented work, rather than the broader, community-driven support that people often require. Moreover, this shift toward formalization has inadvertently excluded older and less literate community members, as volunteers are now expected to read and write proficiently to be able to complete standardized records and reporting forms.

Given that international organizations and palliative care advocates are encouraging the integration of community-based palliative care into formal healthcare systems, it is important that we know more about the experience and challenges of palliative care formalization. This paper focuses on a case study from Jakarta, the capital of Indonesia, where palliative care is currently still developing and community-based care is facing potential formalization (Ind: *formalisasi*), through a process of structuring and institutionalizing the volunteer practices that currently focus primarily on caring for patients with cancer. Specifically, this paper addresses two main research questions: (1) How do individuals involved in community-based palliative care define formalization and how do they perceive the movement toward formalization? (2) What opportunities and challenges are associated with the formalization of community-based palliative care?

Where most publications on this topic take the form of synthesis, editorial, and review articles,^13^ the findings presented in this paper are grounded in empirical data, which still remain relatively limited in palliative care research, particularly in the Global South.^39^

## Community-based care in Indonesia

Community-based palliative care in Jakarta emerged within a national context of palliative care development that had already been underway for more than two decades. While hospital-based palliative care began in Indonesia in the early 1990s through the work of palliative care specialists,^40–42^ community-based approaches were primarily driven by non-governmental initiatives that focused on cancer care. Community-based palliative care initiatives have developed earlier in the Indonesian city of Surabaya in 2010. That initiative started by recruiting 50 female community volunteers (primarily housewives) for palliative care training.^47^

In 2015, the Jakarta branch of the Indonesian Cancer Foundation (ICF) and the NGO Rachel House collaborated with the District Health Office, with support from the Singapore International Foundation, to train female community health volunteers (*kader*) in palliative care, alongside healthcare professionals.^43,44^ Volunteers who successfully completed the training were called community-based palliative care volunteers (*kader paliatif*). Both ICF and Rachel House had already been providing palliative care services in Jakarta prior to this collaboration. ICF, a semi-governmental organization, has offered home hospice care for cancer patients since 1995.^43^ Rachel House has pioneered children’s palliative care in Indonesia since 2006, providing support for children with life-limiting conditions such as cancer and HIV, particularly those from low-income families.^45^

The female community health volunteers invited to join the *kader paliatif* program in 2015 were members of the Family Welfare Program (*Pembinaan Kesejahteraan Keluarga*, or PKK). The activities of the PKK reflect what Suryakusuma^46^ conceptualizes as “state *ibuism*”—a gender ideology that institutionalizes women’s duties to serve their families, husbands, communities, and the state. These invited volunteers were typically homemakers or informal workers (e.g., city cleaners, small food stall owners, or food delivery drivers) who were already involved in broader community health initiatives such as maternal and child health, nutrition, and disease prevention programs.

The long-term vision of this initiative is to integrate community-based palliative care into the existing public health framework, following the model of earlier programs dating back to the 1970s, when the government launched the national family planning program. Since then, local neighborhood volunteers have played a vital role as intermediaries between health services and communities.^43,47^ Another notable example is the maternal and child health volunteer program, which remains Indonesia’s largest and longest-running community-based health initiative, involving approximately 1.5 million volunteers.^48,49^ Drawing on the success of these earlier programs and their proven scalability at the national level, those who initiated the first community-based palliative care initiatives seek to adopt a similar approach.

Between 2015 and 2022, the ICF trained 611 volunteers in palliative care, of whom approximately 100 received advanced training.^43^ The training program aimed to strengthen the role and capacity of community-based palliative care teams by improving their knowledge of cancer and their understanding of how to support patients throughout the trajectory of the illness. This included early detection initiatives (e.g., promoting cancer awareness in their neighborhoods and facilitating access to cervical and breast cancer screenings) as well as post-hospital discharge support. Volunteers were trained to provide not only basic physical care but also emotional, social, and spiritual assistance for patients and their families. The advanced training program enhanced volunteers’ practical skills in managing common symptoms such as difficulty with eating, shortness of breath, wounds, lymphadenitis, and pain. Trained palliative care volunteers were expected to coordinate with and report to both the primary healthcare centers (*puskesmas*) and the ICF when assisting those in their community who were experiencing health-related difficulties at home.

Despite the comprehensive training programs aimed at supporting the professionalization of volunteers, in practice, structural and institutional support for delivering community-based palliative care remains limited. First, there has been a lack of recognition in policy of the role of palliative care volunteers within both the national palliative care and broader community health frameworks.^50,51^ It was not until December 2023 that the term *kader paliatif* (palliative care volunteer) was formally mentioned in national palliative care policy, acknowledging the role of volunteers as an integral part of both primary and hospital-based palliative care.^27^

Second, because the training was developed and implemented by NGOs rather than through the public health system, many health professionals, including those working in hospitals and primary care centers, remain unfamiliar with the role of palliative care volunteers. The scope of volunteers’ activities also varies widely and tends to be flexible, depending on individual availability and resources. In the area where the fieldwork was conducted, as well as in many others (according to the volunteer coordinator and NGO staff), community-based palliative care has yet to be formally integrated into the primary healthcare structure. There is little systematic coordination with the local health clinic (*puskesmas*), and only a few volunteers actively report cases to the ICF or accompany ICF doctors and nurses during home visits.

As one NGO staff member explained, because their organization only provides training, they cannot and should not impose obligations on volunteers to fulfill all the “tasks” outlined during training. Without formal institutional integration into the primary healthcare system and in the absence of a dedicated funding mechanism, community-based palliative care in Indonesia remains largely dependent on voluntary efforts and personal commitment.

## Methodology

### Study setting

The study was conducted in Jakarta, the capital city of Indonesia, where most of the country’s tertiary hospitals are located. Situated on the northwest coast of Java, Jakarta has a population density of approximately 22,000 people per square kilometer. According to the latest United Nations report, released in November 2025, Jakarta is the world’s most populous city, being home to 42 million people.^52^ As a metropolitan area, Jakarta serves as Indonesia’s central hub for politics and the economy. The cityscape presents a striking contrast, with modern skyscrapers standing alongside densely populated *kampung* (urban villages). Although the city contributes about 17% of Indonesia’s total Gross Regional Domestic Product, approximately 450,000 people in Jakarta live below the poverty line. Administratively, Jakarta consists of five districts and a group of islands.^53,54^ The volunteers observed in this study were mostly based in one of these districts, which is predominantly composed of low-rise urban villages that are home to residents from lower socio-economic backgrounds.

### Method

This paper is based on an ethnographic study of palliative care provision in Jakarta. Ethnography is a research approach in which the ethnographer studies a specific social or cultural group to gain a deeper understanding of it by actively participating in the group’s activities over a longer period of time, in order to develop an insider perspective and experience social life as members do.^55^ This study is part of a larger research project titled Globalizing Palliative Care, which investigates the globalization and cultural mediation of palliative care practices, policies, and discourses.^56^ Fieldwork in Jakarta was conducted by H.A. over 8 months in 2022, with a follow-up visit in 2023. Data collection was carried out through a combination of qualitative methods, including participant observation,^57^ semi-structured interviews, two focus group discussions with palliative care volunteers, and document analysis.^58,59^ The ethnographic fieldwork took place in a community-based palliative care volunteer program, patients’ home, as well as in the palliative care unit of a tertiary hospital. This paper presents an analysis of the data collected from the community-based palliative care program.

Participants were eligible for inclusion if they were 18 years of age or older, had completed community-based palliative care training, were currently serving as community-based palliative care work in Jakarta, and were able to provide informed consent. Individuals were excluded if they had never served as community-based palliative care volunteers or if they had cognitive impairments or communication barriers that would prevent their participation. The number of participants in this ethnographic study is not determined through statistical calculations but through the richness, variation, and sufficiency of the data collected.^60^ Using purposive sampling, we select participants based on the relevance of the insights they can provide rather than the quantity of cases.^61^ Instead of aiming for a predetermined sample size, we prioritize prolonged engagement to achieve in-depth information and understanding.^60^ The recruitment of volunteers and NGO staff members began through an initial contact at the NGO, after which additional participants were identified through snowball approach. Participants provided verbal rather than written informed consent, which is a common and appropriate practice in ethnographic research.^62^ In ethnographic research, consent is understood as a continuous, negotiated process rather than a single moment of agreement.^63^

As part of the fieldwork, H.A. observed the activities of palliative care volunteers during home visits (a total of seven households) and at volunteer gatherings. A.S. also joined to observe one of these gatherings. At these gatherings, volunteers reflected on their work, sharing experiences and challenges with the group. In addition, H.A. co-created a documentary film on community-based palliative care volunteers in Jakarta in 2024.^64^ Detailed written field notes were taken during all observations, and H.A. kept a reflective journal to examine and critically consider her role and positionality.

Semi-structured interviews were also conducted with 14 palliative care volunteers and six staff members from the ICF (including two doctors, two nurses, and two education officers). The volunteers were all over 50 years old at the time of the research. The interviews focused on participants’ backgrounds, roles, experiences, strengths, challenges, and strategies as palliative care volunteers, as well as their visions for the development of palliative care. All interviews were conducted in Indonesian (H.A.’s first language) and audio-recorded, and these were complemented by informal conversations.

To ensure rigor and quality in the study, we employed established qualitative strategies, including the use of multiple data sources by combining semi-structured interviews and participant observations conducted during 8 months of ethnographic fieldwork with the community in Jakarta, as well as by involving two participant groups—palliative care volunteers and NGO workers. H.A. also maintained detailed documentation of observations through reflexive field notes.

### Data analysis

We used a reflexive thematic analysis to examine the data, as it offers a flexible yet rigorous approach to exploring volunteers’ experiences and perspectives. Thematic analysis is useful to exploring lived experiences, examining participants’ perceptions of specific phenomena, and identifying factors such as barriers and facilitators,^65^ making it particularly relevant for examining the formalization of community-based palliative care volunteers and the opportunities and challenges it entails. Within ethnographic research, it is widely accepted that the researcher’s role and positionality influence both how the data gathered and the interpretation of the results.^65,66^ Drawing on H.A.’s long-term engagement with participants during fieldwork, previous experience living and working in Indonesia for over 25 years, and fluency as a native Indonesian speaker helped strengthen rapport with participants. H.A.’s positionalities also shaped how themes were identified and how meaning was constructed during the interpretation of the findings. These emerging themes were discussed with A.S. and N.L.D. during regular team meetings held throughout the fieldwork period and after its completion. During the meeting the data were revisited to strengthen the analysis. Throughout the iterative process of the analysis, we moved repeatedly between the data, codes, and emerging themes, refining interpretations as new insights developed. To enhance the analysis, we also presented the preliminary findings at the 2024 Public Health Palliative Care Conference, which provided us with valuable feedback for refining the themes.

## Results

This section explores the definition of the formalization of community-based palliative care and the opportunities and challenges identified by the palliative care volunteers and NGO staff members.

### Definition

In the first meeting with the education officer who developed the curriculum for the palliative care volunteer training, she emphasized that volunteer practices in Indonesia differ from those in many other countries, particularly those in “the West,” where volunteers are typically structured, organized, and mobilized by institutions such as charities, religious organizations, or even government bodies. In contrast, volunteer activities in Indonesia, she said, tend to be informal and unstructured, emerging from bottom-up initiatives that are deeply rooted in cultural and religious values. After that meeting, H.A. soon discovered that the palliative care volunteers and the staff members from the NGO that provides their training have multiple interpretations of what the formalization of palliative care volunteer activities might entail.

When NGO workers and palliative care volunteers referred to “formalization” (*formalisasi*), they indicated a range of things that this could entail, for example, payment or other financial compensation, formal recognition, or wearing a uniform. Often, their notion of formalization was informed by the roles of other “formalized” community-health volunteers in Indonesia, such as the mother and child care volunteers. Among the volunteers interviewed, some viewed formalization as an opportunity to receive financial incentives for their work, similar to volunteers in other public or community-based health programs. Others, however, understood formalization as more than just the possibility of receiving a financial reward, describing it as a form of recognition and appreciation for their contributions as volunteers. These volunteers saw formalization as a way to enhance the legitimacy of their role and to strengthen their partnerships with healthcare professionals, thereby improving support for patients. Among the group of volunteers whom I interviewed a variety of perspectives on formalization were expressed (sometimes even coming from the same ambivalent person).

### Opportunities

In this section, we discuss two opportunities that might arise from formalizing palliative care volunteer programs from the perspective of the volunteers—namely, legitimacy and recognition, and financial rewards.

#### Legitimacy and recognition

Volunteers point out that formalization of their role may bring more legitimacy to and recognition of their work. Involvement in community organizations—where their role is acknowledged as part of the healthcare structure—provides community health workers with a sense of social standing and pride. One palliative care volunteer, Mrs. Evi, who is also involved in a maternal health program, observed:

My husband and children are proud of my role as a *kader* (community health volunteer) and the contribution I make to our community.

However, the volunteers also expressed a desire for their social standing to be more clearly and publicly recognized as part of the formal healthcare system. For example, one palliative care volunteer, Mrs. Narti, was almost kicked out of the hospital when accompanying a patient because she did not have a uniform or identification to indicate her status as a volunteer.

One day, I was at the hospital, accompanying a patient and waiting in the line to get an appointment scheduled for them. A security staff member approached me, asking many questions . . . He (the security person) thought that I was a *calo* (a broker), like others in the hospital who charge patients a lot of money to handle this kind of task. He almost kicked me out (*mengusir)* (of the hospital).

Similar experiences of being mistaken for *calo* were also shared by other palliative care volunteers and were discussed during one of the volunteer gatherings H.A. attended. This unpleasant experience motivated Mrs. Narti and other volunteers to advocate for the formalization of the palliative care volunteer role. She hoped that, as a result, volunteers would be officially recognized within the healthcare structure and treated with respect. For her, formalization included having a uniform and volunteer identification. The uniform, in this context, would not only be functional in distinguishing volunteers from *calo* but would also have a symbolic value. In Indonesia, as in many other contexts, a uniform is a source of social status in and of itself.

#### Financial reward

On the way to visit a patient, while H.A. was riding pillion on 58-year-old Mrs. Susi’s motorbike, she told H.A. how essential the motorbike was to her work. Although it was old and often broke down, it remained indispensable for supporting patients in her neighborhood. Because her neighborhood is quite large, she relies on the motorbike to reach patients in different parts of the community. She also depends on it to accompany patients to hospital and to collect medications from the NGO. “But if there’s no petrol, the motorbike can’t move, right?” she said, laughing behind her face mask. Mrs. Susi raised concerns about transportation costs, which have been a persistent challenge to her volunteer activities. As palliative care volunteers receive no financial incentives or reimbursement for transportation, she has to cover the costs herself, occasionally seeking small donations from the village head when she runs out of money.

Volunteers’ sense of duty and generosity were also made clear through other acts of giving. Bringing something—such as food, to home visits, though not mandatory, is often regarded as a social obligation. During one interview, Mrs. Dani explained that she once postponed a home visit because she could not afford to buy “something” for the patient’s family, and she did not want to conduct a home visit empty-handed. “I felt bad visiting without bringing anything,” she said. “So, I waited until I had a little money.”

Formalizing community-based palliative care could help volunteers address financial barriers to their work and improve their capacity to support patients—for example, by covering transportation costs for home visits, accompanying patients to hospitals, or collecting medications, and by providing essential supplies for families without volunteers having to rely on personal funds. One volunteer, Mrs. Hana, used a metaphor to describe the necessity of formalization: “There’s an abundance of rice out there, but we don’t have a bowl to hold it. The bowl is important.” She explained that having an organized, structured, and formalized system would enable volunteers to access funding opportunities from public and private organizations and expand community-based palliative care within the broader healthcare system—similar to how volunteers in other community-based health initiatives receive incentives or small salaries.

Furthermore, participants suggested that formalization might help attract new palliative care volunteers, particularly younger community members. Many volunteers noted during one of the meetings that palliative care is not seen as being as “attractive” as other community-based health programs. One of the contributing factors is the lack of financial incentive. Strengthening formal recognition and support structures could therefore help sustain the *kader* workforce.

### Challenges

Volunteers also identified some potential challenges that may arise from formalization. Key among these were concerns over its impact on the spiritual and altruistic character of volunteer work, the increased bureaucratic responsibilities, changes to existing care practices, and having to navigate the language of palliative care.

#### Impact on the spiritual and altruistic character of volunteer motivation

One of the volunteers’ main concerns with formalization centered on balancing religious values with the expectations of formal and professional care work, particularly when financial or material compensation might be involved.

Many volunteers described their motivation using terms such as *suka* (doing something because they enjoy it), *rela* (a willingness to serve without expecting anything in return), and *ikhlas* (sincerity and wholehearted devotion). For example, during a large gathering, which also served as an advocacy event attended by the sub-district head, a district representative, and a physician, one volunteer, Mrs. Amina, delivered a motivational speech to over 50 participants. She said: “Palliative care is *ladang ibadah* (a field of worship) or all of us. We do it *lillahi ta‘ala* (for the sake of Allah), with *ikhlas* (sincerity and wholehearted devotion).” She closed by asking, “And we will be rewarded by Allah, right?,” addressing all the palliative care volunteers at the meeting. The room echoed with a collective “Amin (amen).”

In addition to religious motivation, community-based palliative care work is also shaped by the moral expectation of helping one another within the community. For many volunteers, offering support to neighbors in need, especially those who are ill, is part of a more commonly experienced social and moral obligation. Helping and engaging with neighbors is regarded as a core aspect of social life in Indonesia, reflecting the traditional value of *gotong royong*—a principle centered on solidarity, shared responsibility, and mutual aid. As one interlocutor explained:

When we run out of sugar, we knock on our neighbor’s door and ask for some. It’s the same when someone has a medical emergency and needs to go to the hospital. It’s not the ambulance we call first, but our neighbor. The ambulance takes too long because of the traffic. We rely on ourselves and our community.

On a separate occasion, while we were traveling together on a bus, Mrs. Hana shared her ambivalence about formalization. She worried that introducing financial incentives or formal employment might undermine the altruistic and spiritual motivations that sustain their volunteer work. This fear of losing the grassroots spirit of palliative care volunteering was echoed by others. For instance, Mrs. Amina expressed concern that formalization might shift the focus from simply “being there” for neighbors to meeting targets and performance indicators set by programs.

#### Bureaucratization

Alongside concerns about how the motivation behind volunteer work might change, some volunteers were concerned that formalization would impose new bureaucratic obligations—through paperwork and data reporting—and potentially exacerbate hierarchical tensions between volunteers and salaried health professionals. In one of H.A.’s interviews, Mrs. Cindy, who had been working as a community health worker for over 20 years, shared her experience of being a “formal volunteer” in several community health programs.

“Formalization also means more forms, more reports, more documentation,” she said, pointing to a stack of papers beside the television in her living room. “The health workers at the primary care center often ask us to send the reports suddenly, with very little time to complete them—and sometimes not very nicely. If they ask, we’ll do it—it’s our task to collect the data in the field. But please, ask nicely.”

As Mrs. Cindy highlighted, formalization not only increases reporting responsibilities but can also create friction with healthcare workers, as the coordination structure often positions volunteers as subordinates to primary care staff.

Formalization might also risk excluding some long-standing volunteers if certification becomes a requirement. Many of the current volunteers have been supporting people in their neighborhoods long before palliative care training was introduced. For example, 65-year-old Mrs. Azizah, a volunteer and breast cancer survivor who hosted one of the volunteer gatherings that I attended, explained that she no longer conducts home visits: “I’m not as agile as I used to be. That’s why I can only organize events like this at my house. I still want to support patients and their families,” she said with a smile.

#### Changing care practices

In addition to the potential burden of bureaucratic demands, volunteers and NGO staff worried that formalization might change the nature of caring practices. The changes that they were worried about had at least two dimensions: flexibility in terms of time and the freedom to broaden the target group of care.

Expressing her concern about what she called the *formalization movement*, an NGO staff member used the striking metaphor of the chemical preservative formalin (which is used to keep things from decaying):

Something that has been formalin-ed becomes stiff and rigid—and that’s exactly what we don’t want to happen to the palliative care volunteers.

Most volunteers do not practice palliative care on a regular schedule. They often balance their volunteer work with other responsibilities at home or at their paid jobs—including, for example, Mrs. Fani, who delivers food for a living, and Mrs. Susi, who runs a *warung* (a small food stall) while caring for her ill mother at home. The volunteer work currently has no strict structure or target (e.g., a required number of patients to visit or assist each month). If new systems were to introduce fixed schedules, reporting requirements, or performance targets, volunteers could lose the freedom to organize care around their own rhythms of work and family life. It would also potentially discourage new volunteers from joining.

Volunteers were also worried that defining palliative care too narrowly might exclude certain forms of support for patients who do not have life-threatening illnesses. As Mrs. Cindy and Mrs. Amina pointed out, the current flexible approach allows them to provide care for neighbors with a range of conditions, such as leprosy or mental health issues. Mrs. Amina told me about an occasion when she and her husband had driven a patient with leprosy to the hospital at night. Mrs. Cindy offers another example: she supports her neighbor with mental health issues and had helped connect them to mental health services. These patients had been neglected by their family members and avoided by their other neighbors. For Mrs. Cindy and Mrs. Amina, narrowing the definition of palliative care to clinical categories would risk overlooking the everyday realities of illness and vulnerability in their communities. Their understanding of care goes beyond medical intervention or diagnosis; it encompasses companionship, emotional presence, and the moral obligation to help those around them.

#### Navigating the language of palliative care

The implications of the formalization of palliative care in the community extend beyond administrative structures to the moral and linguistic dimensions of care. If community-based palliative care initiatives become formalized, it will also require volunteers to use the term palliative care more explicitly in their work, through the completion of forms or asking for patients’ or caregivers’ signatures on documents that would force volunteers to use the term “palliative care.” In practice, some volunteers currently deliberately avoid using this term when visiting patients. For example, instead of introducing herself as a palliative care volunteer, Mrs. Rana prefers to refer to herself simply as a community health volunteer from the neighborhood. She worries that using the term palliative care might “complicate things” for patients and their families—not only because they are often unfamiliar with the concept but also because the term can evoke fear and may change the relationship. Many people associate palliative care with death, dying, or even euthanasia, which could create anxiety.^67^ For volunteers like Mrs. Rana, who choose their words carefully to avoid causing harm or fear, formalization could put them in a difficult position, forcing them to use terminology that may frighten those they aim to support.

## Discussion

This paper has sought to create an understanding of the complexity, multiple perspectives of, and ambivalence toward formalization. The findings demonstrate that formalization is not a fixed concept; rather it shifts depending on the actors asked about it. Palliative care volunteers described formalization in ways ranging from introducing visible markers (e.g., uniforms or identity cards) to establishing institutional features such as financial incentives, recognition within the health system, and administrative structures. Volunteers’ views of formalization were shaped by their experiences in both palliative care and other public health programs they had previously engaged with or continued to participate in, such as maternal and child healthcare.^68^

In this paper, we also highlighted some opportunities that might arise from the formalization of community-based volunteer work. Similar to what has been observed in the HIV/AIDS and public health programs in Western Kenya and Tanzania,^32^ some volunteers in this study pointed out that formalization promises greater recognition within the healthcare system, positioning community-based palliative care volunteers as legitimate partners rather than informal or inferior actors. This recognition would include the introduction of significant identifiers, such as uniforms, which are not only necessary to avoid being seen as a *calo* (broker), but are also, in Indonesia and many other places, a source of social status, professional identity, and proof of credibility.^69–71^ This recognition may enhance volunteers’ visibility and appreciation within healthcare structures, particularly when supporting patients in hospital settings and coordinating with local health clinics.

Formalization could also enhance the financial support available to volunteers. This is particularly important given that volunteers often spend money out of pocket to assist others, despite sometimes being in positions of need themselves. In line with what other studies of palliative care volunteers in the Global South, such as those based in Brazil, India, or Rwanda, have shown, palliative care volunteers in Indonesia support their neighbors not only by focusing on illness but also by responding to other related needs, such as providing food or essential household supplies.^23,71–73^ Formalization that includes the provision of financial incentives might also help to sustain the program and attract new palliative care volunteers, particularly younger community members.^74,75^

Despite the opportunities formalization may offer, it also presents significant challenges. In this study, we have highlighted the ambivalence toward the possible changes to volunteer motivation as community-based palliative care becomes formalized. This resonates with other research that has shown that the commodification of informal or unpaid volunteering may alter the volunteering experience.^7,25^ As in Kerala, India,^7,23^ and many countries in Africa,^75^ community-based palliative care in the Indonesian context reflects a strong cultural expectation of mutual aid, where supporting and being present for neighbors is regarded as a fundamental aspect of community life and unfolds organically.^47^ It is deeply rooted in the cultural value of *gotong royong* (mutual aid and cooperation).^76^ This reflects what Thornton et al.^77^ describe as “community logic,” which encourages actors to prioritize shared values, practices, and collective pursuits, fostering trust, reciprocity, and moral responsibility. Furthermore, similar to Suresh Kumar’s findings on palliative care volunteers work in Kerala, India,^7^ volunteers involved in community-based palliative care programs in Jakarta are largely motivated by religious values (*ibadah*), believing that their good deeds will be rewarded in the afterlife. For these religious and cultural reasons, volunteers may be ambivalent about the potential impact of formalization on volunteering motivation, particularly when unpaid voluntary activities become commodified. Volunteers were concerned that introducing financial incentives or formal employment might undermine the altruistic and spiritual motivations that sustain their voluntary work.^78^

Another potential unintended consequence of formalization that concerns volunteers is the increase in bureaucratic obligations (e.g., paperwork and data reporting). The growing emphasis on efficiency, transparency, accountability, and monitoring systems has contributed to the widespread adoption of standardized working practices and formalization in volunteer work.^28^ This is particularly common in voluntary work in the health sector.^25^ The increasing demand for reporting and administrative tasks may exclude people, especially older adults such as Mrs. Azizah, from volunteering. This resonates with an anthropological study in Mozambique on the formalization of home-based HIV/AIDS care volunteers,^36^ which showed how the shift toward formalization excluded older and less literate volunteers as new requirements for record-keeping demanded proficiency in reading and writing.^39^ As the bureaucratic burden of volunteer activities potentially grows through processes of formalization, it may exacerbate intersectional problems, particularly economic and gender inequalities, by shifting care responsibilities to volunteers, most of whom are women.^79^ This dynamic reinforces unequal distributions of labor and time especially across gender.^36^ Moreover, the expanding reliance on volunteer labor carries the risk of reproducing and legitimizing existing power hierarchies within society and the healthcare system, particularly as states and organizations shift responsibilities onto volunteers while continuing to retain authority over the use of resources.

Formalization may also change care practices,^7,25,32,36^ by excluding patients without life-threatening illnesses who still need support, particularly when the scope is narrowed to address only those suffering from specific illnesses. The narrow scope of palliative care in high-income countries may not adequately address the “suffering” of people with life-limiting conditions in many lower-middle income countries.^6^ In the context of limited resources and inequality, community health volunteers often navigate what Alex Nading^35^ describes as “two contradictory forms of medical citizenship: one disciplinary and bureaucratic, the other compassionate and flexible.” These two concepts reflect the work of palliative care volunteers. Although they were trained specifically to provide care for cancer patients, volunteers frequently support individuals in their neighborhoods with a range of conditions, not just cancer. For example, Mrs. Amina and Mrs. Cindy care for their neighbors with leprosy and mental health issues, respectively. This dynamic emphasizes the need to maintain flexibility and community responsiveness within palliative care systems, even as formalization progresses.^6,78,80^

Lastly, one challenge noted that might arise as a result of formalization was related to language—this is a dimension that is distinct from the problems associated with the formalization of other public health programs and is specific to end-of-life and palliative care. When community-based palliative care becomes formalized, volunteers may be required to use the term “palliative care” more frequently. Yet, in Indonesia, as in many other parts of the world, such as Bhutan, the term palliative care is not commonly known.^81^ The term may also emerge as sensitive and contested. Mention of the phrase “palliative care” could therefore potentially harm social relationships, something that has been noticed in other parts of the world as well.^82,83^ Introducing the word “palliative care” may not only cause fear or distress to the patients of community-based palliative care volunteers but may also complicate their relationships.

Suresh Kumar,^7^ in his reflection on community palliative care in India, emphasizes the need to improve the preparatory work that occurs before formalization in order to better understand the social and political dynamics within communities, as well as the factors that facilitate participation in community-based palliative care. As there is an increasing possibility that these practices will be formalized, we offer several recommendations to strengthen the role of community-based palliative care volunteers within the health system. First, formalization should be pursued not as a top-down restructuring but as a co-created process—building partnerships between volunteers and palliative care professionals, rather than hierarchical relationships. As Vijay et al.^23^ note, in the context where specialist care is either unavailable or unaffordable, the strengthening of community and professional care may need to occur simultaneously. Second, attention should be paid to creating administrative procedures that are supportive rather than burdensome, to creating recognition mechanisms that value both formal training and long-standing community experience, and to developing communication strategies that acknowledge the sensitivity of the language of palliative care. Finally, it is important to balance formalization with the spirit of voluntarism. Flexible incentive models could sustain engagement without compromising the spirit, as well as the religious and cultural motivations of volunteerism.

### Strengths and limitations

Given that this research is based on a single case in Jakarta, its findings cannot be generalized to other contexts in Indonesia or beyond. The study focuses mainly on volunteers and NGO workers, and therefore provides limited insights into the perspectives of the patients who were assisted by the volunteers. Future research needs to be conducted to elicit their perspectives. Despite the limitations, this study has several important strengths. Conducted in one of the leading areas for community-based palliative care in the Global South—where palliative care is more developed than in other parts of Indonesia, with its model currently being considered for national rollout—it provides insights that can inform emerging models in similar settings. Moreover, the long-term ethnographic fieldwork underpinning this study allows for a rich, in-depth, and contextualized understanding of how community-based palliative care practices unfold in everyday life.

## Conclusion

Community-based palliative care volunteers hold diverse perspectives on the formalization of their work—a step that is central to integrating palliative care into the public health system. This study shows that volunteers view formalization as happening in various ways, from the introduction of visible markers (e.g., uniforms or identity cards) to the establishment of institutional features (e.g., financial incentives, recognition within the health system, and administrative structures). This study reveals the key opportunities associated with formalization, including improved volunteer legitimacy and recognition in the health system, and enhanced financial rewards. At the same time, this study highlights several challenges, such as concerns over the potential negative impact on spiritual and altruistic motivations, increased bureaucratic responsibilities, changes to existing care practices, and how to navigate the language of palliative care. We suggest that it is important to strengthen volunteers’ roles within the health system without eroding the relational, flexible, and spiritually meaningful aspects of their work. Further research is needed to explore how these dynamics unfold across different regions and cultural contexts and to explore strategies that will maintain the unique strengths of community-based volunteer care when formal structures are introduced.
